# Safety of low-intensity repetitive transcranial magneTic brAin stimUlation foR people living with mUltiple Sclerosis (TAURUS): study protocol for a randomised controlled trial

**DOI:** 10.1186/s13063-022-06526-z

**Published:** 2022-08-03

**Authors:** Kalina Makowiecki, Natasha Stevens, Carlie L. Cullen, Amin Zarghami, Phuong Tram Nguyen, Lewis Johnson, Jennifer Rodger, Mark R. Hinder, Michael Barnett, Kaylene M. Young, Bruce V. Taylor

**Affiliations:** 1 Menzies Institute for Medical Research, University of Tasmania, Hobart, TAS Australia; 2 School of Biological Sciences, The University of Western Australia, Crawley, WA Australia; 3 Perron Institute for Neurological and Translational Science, Nedlands, WA Australia; 4 Sensorimotor Neuroscience and Ageing Research Lab, School of Psychological Sciences, University of Tasmania, Hobart, TAS Australia; 5 Sydney Neuroimaging Analysis Centre (SNAC), Sydney, NSW Australia; 6 Brain & Mind Centre, University of Sydney, Sydney, NSW Australia

**Keywords:** Multiple sclerosis, Brain stimulation, Repetitive transcranial magnetic stimulation, rTMS, iTBS, Remyelination, Myelin, MRI, Anxiety, Safety, Depression

## Abstract

**Background:**

Multiple sclerosis (MS) is an inflammatory and neurodegenerative disease, characterised by oligodendrocyte death and demyelination. Oligodendrocyte progenitor cells can differentiate into new replacement oligodendrocytes; however, remyelination is insufficient to protect neurons from degeneration in people with MS. We previously reported that 4 weeks of daily low-intensity repetitive transcranial magnetic stimulation (rTMS) in an intermittent theta-burst stimulation (iTBS) pattern increased the number of new myelinating oligodendrocytes in healthy adult mice. This study translates this rTMS protocol and aims to determine its safety and tolerability for people living with MS. We will also perform magnetic resonance imaging (MRI) and symptom assessments as preliminary indicators of myelin addition following rTMS.

**Methods:**

Participants (*N* = 30, aged 18–65 years) will have a diagnosis of relapsing-remitting or secondary progressive MS. ≤2 weeks before the intervention, eligible, consenting participants will complete a physical exam, baseline brain MRI scan and participant-reported MS symptom assessments [questionnaires: Fatigue Severity Scale, Quality of Life (AQoL-8D), Hospital Anxiety and Depression Scale; and smartphone-based measures of cognition (electronic symbol digit modalities test), manual dexterity (pinching test, draw a shape test) and gait (U-Turn test)]. Participants will be pseudo-randomly allocated to rTMS (*n*=20) or sham (placebo; *n*=10), stratified by sex. rTMS or sham will be delivered 5 days per week for 4 consecutive weeks (20 sessions, 6 min per day). rTMS will be applied using a 90-mm circular coil at low-intensity (25% maximum stimulator output) in an iTBS pattern. For sham, the coil will be oriented 90° to the scalp, preventing the magnetic field from stimulating the brain. Adverse events will be recorded daily. We will evaluate participant blinding after the first, 10th and final session. After the final session, participants will repeat symptom assessments and brain MRI, for comparison with baseline. Participant-reported assessments will be repeated at 4-month post-allocation follow-up.

**Discussion:**

This study will determine whether this rTMS protocol is safe and tolerable for people with MS. MRI and participant-reported symptom assessments will serve as preliminary indications of rTMS efficacy for myelin addition to inform further studies.

**Trial registration:**

Australian New Zealand Clinical Trials Registry ACTRN12619001196134. Registered on 27 August 2019

## Administrative information

Note: the numbers in curly brackets in this protocol refer to SPIRIT checklist item numbers. The order of the items has been modified to group similar items (see http://www.equator-network.org/reporting-guidelines/spirit-2013-statement-defining-standard-protocol-items-for-clinical-trials/).

**Table:** 

Title {1}	Safety of low-intensity repetitive transcranial magnetic stimulation for people living with multiple sclerosis: study protocol for a randomised controlled trial. **(Acronym):** magneTic brAin stimUlation foR mUltiple Sclerosis (TAURUS).
Trial registration {2a and 2b}.	Scientific title: Phase 1/2 study examining the safety of transcranial magnetic stimulation in people with multiple sclerosis. (Public title: Magnetic brain stimulation for multiple sclerosis trial.) Trial registration: Australian New Zealand Clinical Trials Registry (anzctr.org.au). Identifier: ACTRN12619001196134. Date submitted: 19/07/2019; first registered: 27/08/2019.
Protocol version {3}	V5_01 November 2021
Funding {4}	Royal Hobart Hospital Research Foundation project grant (C0026309). The Menzies Institute for Medical Research (funded Magstim equipment). KM was supported by MS Research Australia Postdoctoral Research Fellowship (19-0696). PTN was supported by Medical Protection Society of Tasmania (MPST) Foundation Grant (PhD scholarship). MRH was supported by an Australian Research Council Future Fellowship (FT150100406). NS, KMY and BVT receive salary support from the Medical Research Future Fund (EPCD0000008). KMY and BVT were supported by a MS Research Australia / Macquarie Group Foundation Paired Fellowship (17-0223).
Author details {5a}	KM, NS, CLC, AZ, PTN, LJ, KMY, BVT: Menzies Institute for Medical Research, University of Tasmania, Hobart, TAS, Australia. MRH: Sensorimotor Neuroscience and Ageing Research Lab, School of Psychological Sciences, University of Tasmania, Hobart, Australia. MB: Sydney Neuroimaging Analysis Centre (SNAC), Sydney, NSW, Australia; Brain & Mind Centre, University of Sydney, Sydney, NSW, Australia. JR: School of Biological Sciences, The University of Western Australia, Crawley, WA, Australia; Perron Institute for Neurological and Translational Science, Nedlands, WA, Australia. * Equal contribution
Name and contact information for the trial sponsor {5b}	University of Tasmania College of Health and Medicine Research Hub Office of Research Services, Private Bag 23, Hobart TAS 7001, Advocate House Level 1, 15 Liverpool Street, Hobart TAS 7000 Clinical.Trials@utas.edu.au +61 3 6226 7592
Role of sponsor {5c}	Funders and study sponsor were not directly involved in study design and will not be involved in collection, management, analysis, or interpretation of data, or the decision to submit for publication. The study sponsor is responsible for the overall conduct of the study, research governance, insurance and indemnity. The sponsor may determine to discontinue the study if it is in the best interest of participants. The sponsor is also responsible for archiving study records and participant data for a minimum of 15 years.

## Introduction

### Background and rationale {6a}

Multiple sclerosis (MS) is a chronic neurological condition; is the leading cause of non-traumatic disability in young adults, affecting over 2.3 million people worldwide; and in 2017, was estimated to cost the Australian community AUD$1.75 billion [1]. MS was long considered an autoimmune disease; however, it has an underlying neurodegenerative pathology that can persist even in the absence of immune cell attack [2]. Oligodendrocytes are the central nervous system cells that produce myelin and provide metabolic support to neurons [3]. Their death during MS pathogenesis leads to the formation of demyelinated brain lesions, neurodegeneration and disability accrual [4]. MS clinical management involves modulating or suppressing the immune system, which can be effective in reducing relapse frequency in relapsing remitting MS, but can also have significant side effects [5]. While some remyelination occurs in people with MS, the degree of remyelination is insufficient and ultimately fails [6]. Preclinical studies indicate that remyelination can rapidly improve altered neuronal function caused by myelin loss, and thus could potentially protect neurons from degeneration and mitigate the accrual of disability in people with MS [7]. The current drugs available for MS management may slow but do not stop disease progression, and no treatments actively promote remyelination.

A series of in vitro and in vivo laboratory studies have shown that increasing neuronal activity can increase oligodendrogenesis and facilitate myelin production [8–10]. Repetitive transcranial magnetic stimulation (rTMS) is a non-invasive way to modulate neuronal activity [11]. rTMS has been used in clinical and research settings for several decades and utilises electromagnetic induction to painlessly stimulate the brain and alter cortical excitability [12]. In our preclinical studies, we delivered low-intensity rTMS daily for up to 1 month to the brain of adult mice. Mice that received rTMS in an intermittent theta-burst stimulation (iTBS) pattern doubled the number of new oligodendrocytes in the stimulated cortex. Increased numbers of oligodendrocytes were already present after 2 weeks of stimulation but they were immature and did not produce myelin. When the stimulation was stopped at this point, the new cells failed to survive and myelinate. However, when stimulation was sustained for 4 weeks, the new oligodendrocytes matured, elaborated myelin internodes and survived after the stimulation ceased [13]. In this study, we translate this low-intensity rTMS protocol and evaluate its safety for people with MS.

### Risks

rTMS is generally well-tolerated and considered safe with common side effects being mild, such as scalp discomfort or tingling during stimulation and headaches or dizziness, which generally subside soon after stimulation. Although extremely rare, other side-effects associated with rTMS include hearing loss due to the noise from the coil and its proximity to the ear, or seizure induction, particularly in those who have epilepsy or other seizure histories [14, 15]. rTMS is most commonly applied at intensities ranging from 80 to 120% of resting motor threshold (~30–65% maximum stimulator output, MSO) using a figure of 8 coil to direct the highest intensity to a focal target region [16]. The lower intensity (25% MSO) and diffuse stimulation by use of a circular coil, markedly reduces risks of side-effects, which increase with intensity of stimulation [15]. rTMS has not been associated with detrimental effects on fatigue, anxiety or depression in people with MS or in healthy populations; therefore, the risk of exacerbating or causing these symptoms appears low.

rTMS has been widely used in clinical trials and research settings, with effects of stimulation depending on stimulation frequency, intensity, region and focality of stimulation and number of sessions [12]. Previous studies applying various forms of rTMS suggest that it is safe in people with MS, and a recent review concluded probable efficacy (level B evidence) of focal iTBS to improve lower limb spasticity (reviewed by Lefaucheur et al. 2020 [16]). Small studies using various rTMS protocols suggest potential benefits of rTMS to treat other MS symptoms including fatigue and depression [17], working memory performance [18] and manual dexterity [19]. This will be the first study to deliver and evaluate the safety of this low-intensity rTMS protocol and collect exploratory data on the utility of rTMS to promote myelin addition to the brain in people with MS.

The principal aim of this study is to determine whether low-intensity rTMS is safe and tolerated by people with MS, when delivered in 20 sessions over a maximum of 5 consecutive weeks. We will use magnetic resonance imaging (MRI) and MS symptom assessments to indicate whether rTMS is able to increase myelin addition or improve other aspects of MS symptomology, which we will use to inform future, larger scale efficacy studies.

## Objectives {7}

### Primary objective

To determine if low-intensity rTMS using an iTBS pattern, delivered across 20 sessions over a maximum of 5 weeks, is safe and tolerable for people living with MS.

### Secondary objectives

- To determine if a 5 days per week treatment regime is feasible for people with MS.
- To determine if the sham protocol is sufficient to maintain participant blinding.
- To determine if there is any evidence that rTMS alters myelin addition as detected using MRI measurements, to warrant further analysis in a phase II efficacy trial.
- To explore if there is any benefit of rTMS to MS symptoms (anxiety and depression, quality of life, fatigue, cognition and motor symptoms).

## Trial design {8}

Randomised placebo (sham) controlled, parallel design with 2:1 ratio of participants in the active rTMS group compared to the sham control (placebo) group. Phase 1 safety, feasibility and exploratory framework.

## Methods: participants, interventions and outcomes

### Study setting {9}

This study will be conducted at a single site: Clinical Research Facility at the Medical Sciences Precinct (MSP), University of Tasmania (UTAS), Tasmania, Australia.

### Eligibility criteria {10}

A person will be eligible to participate if they are age 18–65 years with an MS diagnosis provided by an MS neurologist; have been stable (on or off MS treatment) and relapse free for 3 months, with an Expanded Disability Status Scale (EDSS) between 1.5 and 6; are willing and able to travel to the study site every weekday for 4 consecutive weeks; and are capable of providing informed consent.

A person will be ineligible to participate if they:

(i). Have metal inside their head or body (i.e. cardiac pacemaker) as metal may be affected by the magnetic field generated by rTMS but will certainly respond to the magnetic field generated by MRI.
(ii). Are pregnant or intend to become pregnant, as the known influence of pregnancy hormones on myelination, remyelination and MS relapse [20] could confound results.
(iii). Have a history of seizures, epilepsy, serious head trauma*, significant and ongoing substance abuse*, stroke, brain surgery, bipolar disorder, mania, claustrophobia or uncontrolled migraines* (including receiving tricyclic antidepressants, neuroleptics or antiseizure medication for the treatment of any indications listed here). These exclusion criteria are standard for rTMS studies and are based on theoretical or potential, rather than proven risk. For example, epilepsy, brain trauma and stroke can impact the structure of the neuronal circuitry and can result in people having a lower seizure threshold and could therefore increase the potential risk of seizure induced by rTMS.
(iv). Have an EDSS ≤1 or ≥6.5. This exclusion criteria ensures that all participants are mobile and require no more than a walking cane for support, so are still mobile enough to attend study visits. People with more severe disability have significant neuron loss and chronic lesions, making them less able to benefit from the hypothesised remyelination effects of rTMS. We include people with an EDSS between 1.5 and 6, who likely have less neuron loss and are the most likely responders.
(v). Previously received transcranial magnetic stimulation. Such people will have experienced the sensation associated with rTMS previously. This exclusion criterion is to maintain blinding of the participant.
(vi). English illiterate or limited spoken English proficiency (to enable informed consent, participant reported outcomes and concomitant medication reporting at each visit). Participants able to provide a suitable translator/interpreter for the duration of the study (including phone calls, questionnaires and visits) may be included. Translators do not need to be accredited and can be a suitable friend or family member of the participant.
(vii). Currently involved in another interventional clinical trial.
(viii). Do not have access to a smartphone (to enable cognitive function tests to be completed using the Floodlight Open smartphone app).

An eligibility questionnaire has been developed to screen potential participants based on the criteria above (adapted from [21]. If the answer is “yes” to any of the exclusion criteria, participants will be informed they are not eligible for this trial. Eligible participants will be invited to visit 1 and provided a participant information sheet.

*Note: At the discretion of the clinical PI, participants with previous minor head trauma (e.g. concussion) without surgical intervention may be included if there are no ongoing clinical signs directly attributable to traumatic brain injury. Participants with resolved substance abuse may be included if the substance abuse has been resolved for 5 or more years, and therefore, substance use during the trial would be unlikely. Participants with a history of migraine may be included, at clinical PI discretion, if migraines are well-managed, infrequent (<1 per month) and not complicated, or if migraines have not occurred for at least 1 year before eligibility screening and trial enrolment. This criterion is intended to exclude those who are potentially at a higher risk of migraine adverse reactions to rTMS.

### Who will take informed consent? {26a}

A suitably trained, qualified and delegated researcher will obtain informed consent. Researchers will ensure participants understand what is involved as well as the risks. They will explain that this is a safety and feasibility trial, and that this intervention programme has not been delivered to people with MS before. Participants will be required to be fully informed with all questions answered, and required to provide their written informed consent form prior to any procedure being performed.

### Additional consent provisions for collection and use of participant data and biological specimens {26b}

Consent will also include a statement on future use of data collected. Biological specimens will not be collected. The participants will consent to have their referring clinician/neurologist informed of their participation in the study as well as relevant health outcomes and any serious adverse events that may occur.

### Interventions

#### Explanation for the choice of comparators {6b}

We will compare rTMS to sham (control) treatment groups. Participants are not required to stop concomitant MS treatments while on the study; thus, they will not be deprived of treatment while in this study. Sham control is the most rigorous comparator for assessing the safety or tolerability of the rTMS intervention. An assessment of efficacy is not the primary outcome for this study, but we will assess the capacity of our sham protocol to maintain participant blinding to inform a future phase II study.

#### Intervention description {11a}

Participants will receive rTMS or sham stimulation 5 days per week (weekdays) ideally over 4 consecutive weeks (20 sessions total). A maximum gap of 3 days is allowed between sessions, with all 20 sessions being delivered within a maximum of 5 consecutive weeks. rTMS will be delivered using a Magstim Rapid2 (Magstim Rapid2 package part numbers: 3012-00 Rapid2 Mainframe – 50Hz; 3013-00 Single Power Supply Unit; 3022-00 User Interface; Magstim Ltd, Whitland, UK) and Magstim 90mm standard circle coil (part number: 3193-00, Magstim Ltd. Whitland, UK). Participants will be seated upright in a comfortable reclinable chair, positioned so that the participant cannot see the Magstim Rapid2 user interface screen. The stimulation intensity will be set at 25% maximum stimulator output and parameters set for iTBS pattern: bursts of 3 pulses at 50Hz, repeated at 5Hz for a 2-s period (10 bursts), followed by an 8 sec gap (10-s cycle time), repeated for 20 cycles (600 pulses, 3 min) [22].

Coil target position will be determined relative to the vertex (top centre point of scalp, where sagittal and coronal planes intersect), found using a flexible tape measure and marking the mid-point between the nasion and inion, and pre-auricular (ear landmark) on each side, respectively. From the vertex, the target coil position will be marked with a whiteboard marker at 2cm lateral and aligned with the vertex in the anterior-posterior axis on both the left and right sides of the scalp. For rTMS delivery, the circular rTMS coil will be oriented so that the plane of the coil is tangential to the scalp and the central hole over the target mark. The coil handle will be pointing backwards, at 45° to the scalp midline. By positioning the circular coil in this way, we can stimulate a broad cortical area including frontal and parietal regions, consistent with our preclinical protocol [13]. Stimulation of the left or right hemisphere first will be counter-balanced between participants, but for individual participants will be consistent between sessions. After completing the 3 minutes of iTBS to the first hemisphere, the coil is then flipped and positioned to stimulate the second hemisphere for a further 3 minutes. Flipping the coil between hemispheres ensures that both hemispheres of the brain receive anteroposterior-induced current on the lateral portion of the coil for the second phase component of the biphasic pulse (i.e. side ‘A’ against the scalp of the right hemisphere results in an anti-clockwise current in the coil; side ‘B’ against the scalp on the left hemisphere results in a clockwise current flow in the 2^nd^ phase). Neuronal excitability is sensitive to the direction of the induced current and with lower intensity (below motor threshold) stimulation, only the anteroposterior induced-current direction in the effective (second) component of the biphasic pulse results in enduring excitability changes [23].

For sham, the procedure will be the same except that the coil will be angled at 90° to the scalp surface (i.e. outer edge profile of the coil touching scalp target location), directing the magnetic field perpendicular to the brain, effectively preventing stimulation. This method of sham stimulation has been used previously and its inclusion is listed as a criterion for rTMS trial quality (reviewed in [24]), as it is effective for blinding to intervention allocation in TMS-naïve participants, and ensures clicking sounds and timing aspects of the procedure are consistent between groups.

#### Criteria for discontinuing or modifying allocated interventions {11b}

Participants are free to withdraw from the study at any time. Participants will be withdrawn from the study if they develop any contraindications:

- Become pregnant
- Receive a metal implant
- Develop seizures
- Begin taking tricyclic anti-depressants, neuroleptics/ anti-psychotic or anti-seizure medications;
- Are no longer fit to participate (e.g. MS relapse or change in disease modifying therapy or prescribed high dose steroids) at clinical PI discretion; test positive for COVID-19; or as an urgent safety measure.

The sponsor, Health and Medical Human Research Ethics Committee (HMHREC) or Therapeutic Goods Administration (TGA) may determine it is within the best interest of study participants or the trial to terminate the study and discontinue the intervention for enrolled participants. Written confirmation will be given to the CI. This may be due to but not limited to serious safety concerns, success or failure of the primary outcome, serious breaches, acts of fraud, critical findings or persistent non-compliance that negatively impacts the patient safety or data integrity. If the study is terminated early, appropriate follow-up and care of participants will be arranged.

#### Strategies to improve adherence to interventions {11c}

Participants are required to attend the study site in person to receive the intervention, and any missed sessions are recorded to monitor adherence. To improve adherence, appointment times are flexible between 8am and 6pm each day (room and staff availability permitting), are not required to be at the same time of day and are not required on weekends. To allow flexibility for long weekends, illness or other factors that prevent attending on site, a window of +3 days is tolerated between intervention sessions, with up to 1 additional week (maximum 5 weeks) tolerated to complete the 20 sessions. Free and accessible parking is provided on site. Participants will be reimbursed for time and travel with a $100 voucher at the end of the intervention period.

#### Relevant concomitant care permitted or prohibited during the trial {11d}

Participants are permitted to take current disease-modifying therapies/medications and physical rehabilitation treatment as usual during the trial, except for medications specifically listed under eligibility criteria and criteria for discontinuation sections. Treatment history, concomitant medications at baseline and any changes to medications will be recorded daily during the intervention.

#### Provisions for post-trial care {30}

At the end of the trial, participants will return to usual care of their treating neurologist. If participants suffer adverse or serious adverse events as a result of the trial, they will be treated in the public health care system. The University of Tasmania (UTAS) will provide insurance and indemnity, including legal liability to pay damages as a result of any claim or claims made against the UTAS, a protected person or affiliate during the protection period in consequence of clinical trials.

### Outcomes {12}

#### Primary outcomes

The primary outcome will be the incidence of treatment emergent adverse events (AE) and serious adverse events (SAE), recorded at each visit during the intervention phase. Incidence will be compared between rTMS and sham using the proportion of participants that had at least one AE against participants who experienced no AEs (each participant counted once). AEs leading to premature discontinuation from the study intervention and serious treatment-emergent AEs will be presented either in a table or a listing. A clinically acceptable difference between the rTMS and sham conditions in treatment emergent events is 10% for adverse reactions and 0% for suspected unexpected serious adverse reactions (SUSARs). We defined this clinically acceptable difference based on previously reported rates of the most common side effects in sham-controlled rTMS clinical trials for depression, which were headache (mean incidence rates of 16% sham vs. 28% rTMS; 12% difference) and scalp pain/discomfort (mean incidence rates of 15% sham vs. 39% rTMS; 24% difference) [25]. Clinical trials using rTMS for depression typically deliver higher intensity and longer duration stimulation, so we expect lower incidence rates of these common adverse reactions. Risk of serious adverse reactions is reduced by excluding participants who are potentially at higher risk of serious adverse reactions to rTMS (e.g. history of seizure) [14, 15].

#### Secondary outcomes

- *Protocol compliance and adherence* to treatment schedule between active rTMS and sham control groups, comparing proportion of protocol-compliant and non-protocol compliant participants overall and separated by intervention group.
- *Blinding success* will be measured by asking participants which intervention they believe they received (real, placebo or unsure) immediately after the first intervention, after their 10th session, and after the final intervention. A visual analogue scale (VAS) will also be used to measure how certain participants feel about their selection of intervention group. Number and percentage of participants’ selections will be presented in a table, separated by intervention group.
- *MRI* data will be evaluated from scans collected at baseline (up to 2 weeks prior to the first intervention visit) and post-intervention (within 2 weeks of the last intervention visit). We will compare the mean or median, as appropriate for distribution, for rTMS and sham groups at each time point, and the change between baseline MRI and post-intervention MRI metrics of sham and rTMS intervention groups, including metrics for whole brain, lobe-specific and lesion locations.
  - Lesion analysis: number of new and number of enlarging T2 hyperintense lesions, combined volume of lesion(s) within each lobe.
  - Atrophy analysis: normalised percentage brain volume change from baseline to post-intervention MRI timepoints will be estimated using SIENA [26], part of FSL [27]. For substructures, including white matter, grey matter, peripheral (cortical) grey matter and ventricular cerebrospinal fluid, SIENAX [26], part of FSL-FIRST [27] will be used to determine normalised tissue volumes at each timepoint.
  - Diffusion tensor imaging (DTI) metrics: mean diffusivity, fractional anisotropy, axial diffusivity and radial diffusivity. Considered together, these metrics are indicative of axonal and myelin integrity.
  - Magnetization transfer ratio (MTR) and quantitative T1 mapping (qT1, mean relaxation time) will be used to measure relatively subtle changes in myelin content [28].

DTI, MTR and qT1 metrics will be obtained for tissue types combined, and for each tissue type segmented into: white matter, grey matter, peripheral (cortical) grey matter and T2 lesional tissue.

- *Symptom and quality of life assessments* will be collected at baseline (before the first intervention session), after the last intervention session, and again at remote follow-up (4 months after intervention allocation) will be used to compare change over time between rTMS and sham groups’ mean or median scores in assessments of:
  - Anxiety and depression, using the Hospital Anxiety and Depression Scale (HADS) [29, 30].
  - Overall quality of life, using the AQoL-8D’s health state utilities and dimensional scores [31].
  - Fatigue, using the Fatigue Severity Scale (FSS) [30, 32].
  - Cognitive and motor MS symptoms, using selected assessments from the Floodlight Open smartphone app test battery [33]. To assess cognitive symptoms, we will use number of correct matched pair responses within 90 s in the electronic Symbol Digit Modalities Test (e-SDMT). We will assess motor symptoms as upper limb manual dexterity using the Pinching Test (number of successful pinches within 30 sec) and Draw a Shape Test (tracing error as mean Hausdorff distance across all shapes); and as gait using the U-turn test (turn speed in radians per second, averaged across all U-turns made within the time period) [33].

### Participant timeline {13}

#### Pre-screening

Participants will complete a preliminary screening questionnaire by phone and be provided with the information sheet. We will contact participants after 2 weeks to confirm interest in participation.

#### Screening and enrolment (visit 0)

**Fig. 1:**
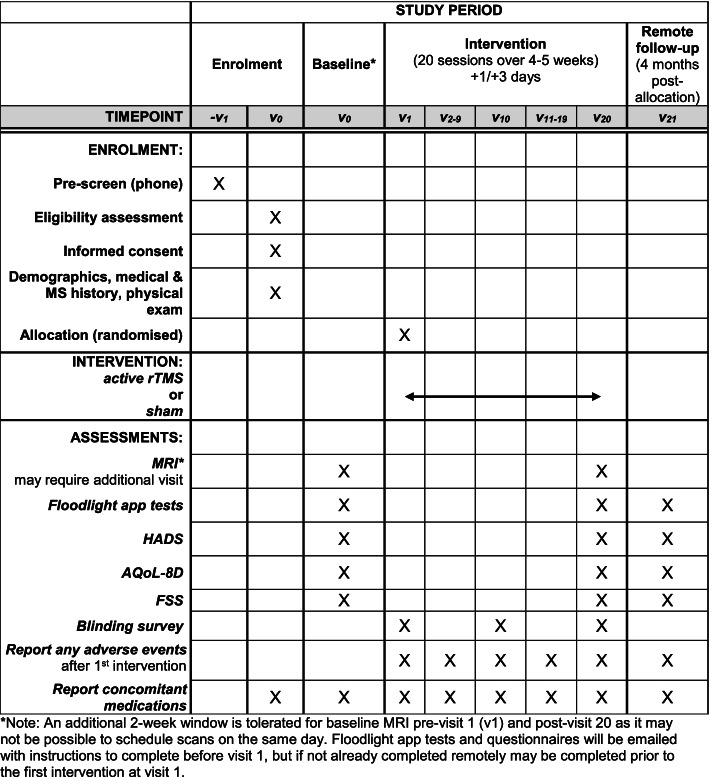
Schedule of enrolment, interventions and assessments

We will collect demographic, medical and MS history data, and participants will be assessed using the EDSS [34] and undergo a physical exam. Medical history will be checked against the clinical records of each participant and eligibility confirmed by the clinical PI. Participants will also register for the Floodlight Open app and researchers will assist them to install the app and login, if needed. The baseline MRI will be scheduled, and the participant granted access to the baseline questionnaires on REDCap (Fig. 1).

#### Baseline MRI

This will be performed ≤ 2 weeks before the first intervention visit*.*

#### Baseline questionnaires

After completing the MRI, participants will be asked (via email) to complete the baseline questionnaires and Floodlight Open app assessments. If baseline assessments were not completed remotely prior to intervention visit 1, these will be completed at visit 1 (prior to the intervention). Group will be allocated prior to the first intervention visit.

#### Intervention phase (visits 1–20) (~15min per day)

Upon arrival each day, participants will be asked to confirm their personal details (name and date of birth) and will be asked if they are experiencing any symptoms that are out of the ordinary. Participants will attend 20 sessions over a period of 4 consecutive weeks. A tolerance of +3 days will be allowed for weekends, sickness or inability to attend a session. Sessions will continue until 20 sessions are complete or up to a maximum of 5 weeks, whichever comes sooner. AEs and any concomitant medication changes will be recorded daily.

#### Blinding success survey (visits 1, 10 and 20)

Participants will be asked which intervention group they think they are in. If they select placebo or real, they will also complete a VAS to assess level of certainty and asked the reason(s) for their guess.

#### Visit 20 only

After the final intervention is conducted, participants will be asked to complete the post-intervention questionnaires (HADS, AQoL, FSS) and Roche Floodlight app assessments (~10 min to complete). Post-intervention MRI (~60 min) will coincide with visit 20, or within 2 weeks of visit 20. Acquisition sequence will be identical to baseline MRI.

#### Remote follow-up (visit 21) (~30–45min)

Questionnaire and Floodlight app assessments will be repeated at 4 months post-allocation and can be conducted remotely via phone with questionnaires returned via REDCap or post, if necessary.

### Sample size {14}

Thirty participants in total will be recruited. The sample size of 30 participants has been selected based on the safety and exploratory nature of the study.

### Recruitment {15}

Patients attending the MS Clinic at the Royal Hobart Hospital will be referred to researchers at the Menzies Institute for recruitment. Menzies researchers will obtain verbal consent to pre-screen participants using a phone screening questionnaire. Based on fulfilment of the eligibility criteria, participants will be sent the participant information sheet for consideration and invited to take part in the study. The participant information sheet will be provided by mail (or in person where practical). Follow-up phone calls will be made 2 weeks after providing documentation if the participant has not contacted the research team. Any questions regarding the study will be answered and their eligibility and agreement to take part confirmed.

Participants will also be recruited by promotion of the trial via the Menzies newsletter, webpage, social media pages or clinic posters/flyers, and local recruitment will be supported by patient advocacy groups such as MS Australia or MS Ltd.

## Assignment of interventions: allocation

### Sequence generation {16a}

After confirming study eligibility, participant allocation to rTMS or sham will be performed automatically using REDCap software. We will use an allocation ratio of 2:1, with twice as many participants allocated to the active rTMS group as sham control, stratified by sex and a block size of 6.

### Concealment mechanism {16b}

Because allocation is performed using REDCap’s randomisation software at the time of participant enrolment, there is no requirement to conceal an allocation sequence. REDCap is user access controlled to maintain blinding of blinded researchers.

### Implementation {16c}

Researchers delivering rTMS will enrol participants and perform randomised group allocation, the allocation sequence will be generated by the REDCap randomisation module. Researchers performing randomisation are necessarily un-blinded to perform the intervention.

## Assignment of interventions: blinding

### Who will be blinded {17a}

Participants will be blind to intervention assignment. MRI raw images will be assessed by blinded researchers. Statistical analyses will be deidentified but unblinded due to the uneven group allocation ratio (2:1); however, researchers performing the statistical analyses will not be involved in participant recruitment, allocation or delivery of the intervention. Researchers delivering rTMS/sham will be unblinded to the intervention group as sham is achieved by changing the orientation of the rTMS coil to prevent the magnetic field from entering the brain. Adverse event data will be collected by the researcher delivering the intervention at each intervention visit and is therefore, also unblinded. Adverse event data will be assessed and graded by the clinical PI who will remain blind to allocations.

### Procedure for unblinding if needed {17b}

It should not be necessary to break the intervention allocation code or unblind the participants during the study period. If necessary, request for un-blinding will be sent to the research PI or CI of the study, and if permission is granted, the allocation revealed. The unblinded researcher performing rTMS or the data manager will be able to access the allocations via the REDCap database software used for randomisation. Only at the end of the study will participants be informed of their allocation group, if requested.

Only after all data queries are resolved and the database is locked will the data be extracted for analysis and the intervention group coded in the dataset.

## Data collection and management

### Plans for assessment and collection of outcomes {18a}

#### Adverse events

AEs will be coded using the Medical Dictionary for Regulatory Activities (MedDRA), each AE will be counted once only for a given participant, presented by System Organ Class (SOC) and preferred term groupings. Details of any AEs will be collected using standardised case report forms and entered electronically into REDCap. The AE start date, stop date, severity, relationship, expectedness, outcome and duration will be recorded. To aid interpretation of AEs and secondary outcome measures, we will also collect data reporting concomitant medications or any changes to medications, recorded daily over the course of the intervention.

#### Protocol compliance

The number of sessions completed will be summed for each participant to assess the proportion of sham vs. rTMS participants that completed all 20 intervention sessions.

#### Blinding success

Participants will be asked by the researcher administering the intervention whether they think they are in the real or placebo (sham) group, or if they are unsure. We will also ask why participants think they are in this group, or why they are unsure, and responses will be entered by the researcher into the electronic case report form (eCRF). If participants select either the real or placebo option, they will also rate their level of certainty of this guess along a VAS by using a slider on a touchscreen from ‘very uncertain’ to ‘very certain’, which is then numerically scored. Successful blinding is defined as a high proportion of “unsure” responses and approximately equal proportion of responses across participant guesses, though participants’ awareness of the allocation ratio could bias guess choice [35]. Blinding will be considered unsuccessful if more than 60% of participants in the sham arm correctly identify their group and rate certainty of their selection in the top 25% of the scale, in line with recommendations from assessments of bias under various (un)blinding scenarios [36, 37]. Blinding success will be primarily assessed after the first intervention visit as this is less likely to be confounded by perceived efficacy of the intervention [38], in accordance with CONSORT 2010 guidelines, item 11 [39].

#### MRI data collection and quality control

MRI will be performed at an accredited facility, by fully trained and qualified personnel at the Royal Hobart Hospital. MRI will be conducted according to the MRI technologist manual for the study. ~20 scans will be performed at each MRI visit, with acquisition sequence - 3D FLAIR, 3DT1, MT on/off, qT1 map and DTI (minimum 32dir). To ensure sufficient quality for analysis (e.g. no movement artefacts), baseline MRI images will be checked by MRI analysts prior to commencing the intervention/ first participant visit, and repeated if necessary. The same quality evaluation will be completed on post-intervention MRI scans. At all stages of the manual, semi-automated and automated analyses of MRI scans, output will be examined by an expert observer (trained clinical trial research assistants, medical doctors and/or clinical neuroimaging specialists) with supervision from Sydney Neuroimaging Analysis Centre (SNAC) directors / image analysis specialists. Where consensus cannot be reached for discrepancies found between analysts, the SNAC Director will make the final determination.

#### MRI image pre-processing for analysis

Prior to the calculation of MRI volumetrics, 3D T1-weighted images will be preprocessed using in-house brain-extraction and lesion-filling tools with manual quality assurance and correction where needed. Brain tissue volumes (whole brain, white matter, grey matter, peripheral (cortical) grey matter, ventricular cerebrospinal fluid), normalised for subject head size, will be estimated at each time point using the SIENAX [26], part of FSL-FIRST methods [27]. Lesion numbers and volumes will be calculated from manually drawn lesion masks on baseline 3D-FLAIR images. Lesion masks will be transformed to follow-up 3D T1-weighted image space and adjusted for lesion activity (see ‘Lesion assessment’ below) by trained analysts.

#### Lesion assessment

A T2-weighted lesion will be identified as a rounded or oval area of hyper-intensity, according to the guidelines by Filippi et al. [40]. A lesion will only be counted once per scan. If a lesion extends across more than one slice, it is only counted once, rather than counting the number of lesions per slice. A lesion will be considered “new” if it was present at the post-intervention MRI and not at baseline. Lesions that are adjacent to a pre-existing lesion but connected to it by a relatively low signal area will also be considered new. Lesions >5mm will be classified as “enlarged” if the lesion size has either increased by at least 100%, or the size has increased in at least two consecutive slices. Lesions <5mm in size must satisfy both criteria to be classified as enlarged.

#### Advanced MRI metric assessments

The diffusion tensor model is fitted to the pre-processed data using FSL-DTIFIT. Magnetisation transfer ratio maps are generated as magnetisation transfer-On image (MTon), co-registered to magnetisation transfer-Off image (MToff) by linear registration. Magnetisation transfer ratio is calculated in voxel-wise fashion as MToff subtract MTon, divided by MToff. Quantitative T1 mapping will be obtained by processing the variable flip-angle spoiled gradient recalled echo data with QUIT open-source software [41]. A map of the longitudinal relaxation time (T1) is generated by linear least-squares fitting of the signal intensity curve as a function of flip angle at constant transfer ratio.

#### Participant-reported MS symptom assessments

Questionnaires will be collected directly into eCRFs via REDCap, minimising transcription errors and missing data (refer to data management section). Paper format questionnaires will be provided to participants if requested.

#### Hospital Anxiety and Depression Scale (HADS)

Fourteen-item questionnaire (7 anxiety items, 7 depression items) with high reliability (test-retest ICC = 0.83; Cronbach’s alpha >0.8) and good criterion validity, with high correlations with other measures of depression and anxiety [29, 30]. Scores ≥8 on each subscale indicate clinically important depression and/or anxiety symptoms and, above this cut-off score, a change of ≥2 points is considered to be clinically meaningful [29]. Use licensed by GL Assessment, MAPI Research Trust, available from GL Assessment [42].

#### Assessment of Quality of Life-8 dimension (AQoL-8D)

The AQoL-8D is a 35-item questionnaire that will assess physical and psychosocial quality of life across 8 dimensions: Independent Living, Senses, Pain, Mental health, Happiness, Self-worth, Coping and Relationships. The AQoL-8D has good test-retest reliability (intraclass correlation coefficient (ICC) = 0.7) and good convergent and predictive validity, as assessed by a multi-instrument comparison study [31]. AQoL-8D has been validated for use in people with MS. [43] Data collection forms and STATA/ SPSS scoring utility algorithm and user manual, and guides for interpreting the clinical importance of subscale difference scores, are available online from [44]. Use registered on 30 May 2020.

#### Fatigue Severity Scale (FSS)

The FSS is a nine-item questionnaire which measures fatigue symptom severity on a seven-point ordinal scale [30, 32]. In an MS population, the FSS has good reliability (ICC = .76, over 6 months) and good-excellent validity metrics (see [45]). A difference of at least 0.45 points is considered a clinically significant change in fatigue [46]. Forms and scoring instructions are available online from [47].

#### Floodlight Open

We will use selected metrics from the battery of tests completed via the Floodlight Open smartphone app [48]. We have selected only the Floodlight metrics with published reliability and validity assessments (e-SDMT, Pinching test, Draw a shape test and U-Turn test) [33]. We may update inclusion or exclusion based on further assessments and validation available at the time of analysis.

#### e-SDMT

Participants will be instructed to match as many pairs as possible in 90 s, by pressing the digit on the onscreen keypad corresponding to the symbol displayed, according to the legend assigning a symbol to each number between 0 and 9. Number of correct matched pair responses has good retest reliability for people with MS (ICC = 0.85) and good concurrent validity (Spearman’s rank correlation with the oral SDMT, *ρ* = 0.85) [33]).

#### Pinching test

Participants will be instructed to use their index and thumb in a pinch-grip to ‘squeeze’ as many on-screen tomatoes as possible within 30 s, and perform each pinch as fast as possible. We will analyse the number of successful pinches, which has moderate retest reliability (ICC = 0.72) and moderate concurrent validity, based on correlation with the 9 hole peg test performance (*ρ* = −0.52), which is the standard manual dexterity assessment used in people with MS. [33]

#### Draw a shape test

Participants will trace the line of 6 shapes, in the direction indicated by an arrow, as quickly and accurately as possible. Each shape is drawn twice, but only the better tracing is used for analysis of tracing error (Hausdorff distance). This test has good retest reliability ICC = 0.85 and moderate correlations with the 9-hole peg standard dexterity test [33]. We will also analyse normalised Hausdorff distances for the 3 curvilinear shapes (spiral, figure-8, circle), which reportedly have greater discrimination between healthy controls and people with MS compared to straight line shapes [49]. Participants will be instructed to use the same hand for all three timepoints in this study for both the pinching test and the draw a shape test.

#### U-turn test

Participants will be instructed to walk at their normal pace between two points 2–4 m apart and complete at least 5 walking turns within 60 s, with their phone in their front or back hip pocket. Mean U-turn speed has good retest reliability (ICC = 0.83) and moderate concurrent validity (correlation with the timed 25-foot walk standard clinical measure, *ρ* = −0.52) [33].

The Floodlight Open smartphone app can be downloaded from Google Play for Android or Apple store for IOS. At the time of writing, the Floodlight Open app is available but will be replaced by Roche Floodlight MS app in the future.

### Plans to promote participant retention and complete follow-up {18b}

If a participant misses more than 3 consecutive intervention visits, they will be considered protocol non-compliant. These participants will be permitted to continue in the study until the end of the 5-week intervention phase, and remain in the study to complete follow-up safety and PROMs, but will be excluded from the follow-up MRI. Outcome measures are collected at the final intervention visit (face to face) and then again 4 months after group allocation. An automatic email is sent to remind participants to complete PROMs via REDCap. Additionally, participants will be contacted three times by two different methods before they are considered ‘lost to follow up’.

Protocol deviations will be monitored monthly by the trial management group and appropriate corrective and preventative actions taken to reduce incidence of protocol deviations. The statistical analysis plan will include per protocol analysis and intention to treat analysis for AE and participant-reported outcomes. As the follow-up MRI will only be conducted for participants without substantial protocol deviations, intention to treat analysis is not possible.

### Data management {19}

REDCap will be used to manage the research data. REDCap is an open source, free, mature, secure web application for building and managing online surveys and databases. It is a robust system for research data management, supporting participant tracking, randomised group allocation, web-based questionnaires, separation of identifying and clinical data and tracking/auditing of data changes. REDCap is fully compliant with good clinical practice (GCP) guidelines, and international standards for clinical trial data management. All research staff will complete training in REDCap prior to entering live study data.

All participant-reported data will be collected directly to eCRF, minimizing transcription errors and missing data. Data validation rules will be used to query potential data errors. All screening and enrolment data will be quality assessed by a second member of the team prior to clinical PI sign off and confirmation of eligibility for group allocation. Clinical PI sign off will be required for all AEs and participant withdrawals. If participants request a paper questionnaire be provided, this will be printed from REDCap and then entered by study personnel at a later date. Quality control will be performed on all data that is not directly entered. Source data verification will be performed against the digital medical record (DMR) for demographics, MS diagnosis, MS type, MS history, MS relapses, MS treatments, EDSS score, general medical history, symptoms and concomitant medications.

The MRI data will be analysed at SNAC and the Menzies Institute for Medical Research, Hobart, according to a pre-defined data dictionary and analysis plan. All MRI scans will be codified and transmitted to SNAC via a secure web portal, according to the MRI transfer manual, for central analysis.

Cognitive and motor assessment data will be directly entered by participants into the Floodlight app. Participants will consent to share their Floodlight ID to enable access to this data from the Roche open access data portal [48]. This data will be downloaded in CSV format and integrated with the study datasets. Floodlight data is stored on Amazon Cloud and data managed by F.Hoffmann La-Roche Ltd.

### Confidentiality {27}

Data will be collected, handled and stored in accordance with GCP guidelines [50], The Privacy Act (1988) [51] and the Australian Code for the Responsible Conduct of Research, 2018 [52]. A data management plan outlining the collection, handling, storage, security, access controls and archiving of study data will be developed in accordance with the sponsor Management of Research Data Procedure.

Identifiable data (name, address, phone number, email, sex, date of birth, emergency contact and emergency phone number, neurologist name and neurologist phone number) collected during screening will not leave the study site. Each participant will be given a unique, pseudonymised participant identifier number that will be used on all case report forms and stored separately from their identifiable data. The participant ID will be assigned sequentially.

Trial data will be made available to suitably qualified members of the study team, monitors and auditors, HMHREC and TGA as far as required by law. Study records will be archived for a minimum of 15 years following study completion, in line with sponsor requirements and Good Clinical Practice guidelines.

Consent will be sought from participants to allow MRI and clinical data to be retained indefinitely and made available for future ethically approved research by us and external collaborators with appropriate confidentiality and data sharing agreements in place.

### Plans for collection, laboratory evaluation and storage of biological specimens for genetic or molecular analysis in this trial/future use {33}

N/A.

## Statistical methods

### Statistical methods for primary and secondary outcomes {20a}

The methods of analysis will be detailed in a statistical analysis plan and signed off by the CI prior to data lock and data analysis taking place.

#### Primary outcome—adverse events

A Fisher’s exact test will be used to compare the proportions of people in sham vs. rTMS who experience any treatment-emergent AEs. If different categories of treatment-emergent AEs are reported, we will run additional tests to compare proportions of AEs of each severity level (treatment emergent AEs, high intensity AEs, SAEs and SUSARs).

#### Secondary outcomes

##### Protocol compliance

We will use a Fisher’s exact test to compare sham and rTMS proportions of participants that are compliant (complete all 20 sessions within the time frame) vs. non-compliant (fail to complete all sessions within the time frame).

##### Blinding success

We will present summary descriptive statistics, frequency (*n*) and percentages per guess category, and median certainty scores (VAS), cross-tabulated by intervention group, at each time point.

##### MRI

Proportion of participants with new lesions or enlarging lesions at the post-intervention time point will be compared between sham and rTMS using a Fisher’s exact test. Continuous MRI measures will be analysed with full factorial mixed MANOVA [within-subjects factor: time (baseline, post-treatment); between-subject factor: intervention group (rTMS, sham)], examining main effects and time*treatment interaction. If assumptions of MANOVA are violated when analysing combined MRI measures (e.g. items too highly correlated; Box’s test violated), we will split up the analyses into variable sets by tissue type and region (e.g. whole brain, lobe-specific measures separated). We may use deidentified data from publicly available MRI databases, including healthy control participants, in preliminary analyses to inform variable sets in multivariate analyses and to aid interpretation of results.

We will follow up MANOVA(s) with canonical discriminant analysis to examine rTMS/sham group separation on the linear combination of measures of myelin. We will also run univariate mixed-ANOVAs on measures of total myelin content, to examine the simple effects of intervention group at the post-intervention time point, as detailed a priori in the statistical analyses plan. False positives from multiple comparisons will be controlled using the false discovery rate (FDR) procedure [53] with threshold set at the conventional 5% level.

##### Participant-reported questionnaire and Floodlight data

Continuous outcomes (AQol8D, HADS, FSS, U-Turn speed and Draw a Shape tracing error) will be compared between rTMS and sham conditions with univariate mixed-ANOVAs, as described above for MRI. Significant time by intervention interactions will be followed up with simple effects tests, comparing rTMS to sham group means within each time point.

Outcomes with count data (e-SDMT, Pinching, tracing error in the Draw a Shape test) will be analysed using Mann-Whitney *U* test to compare medians between rTMS and sham groups within each time point. The time by intervention interaction will also be examined using Mann-Whitney *U* tests on within-subject change from baseline, separately for the post-intervention and follow-up timepoints, controlling for false positives using FDR set at 5% threshold.

The statistical analyses carried out may be modified based on distributions observed and test assumptions. For all statistics, the level for significance will be set at *p*<0.05. Summary statistics will be prepared with means and standard deviations considered for normally distributed outcomes or median and interquartile range for non-normally distributed outcomes, as appropriate.

### Interim analyses {21b}

No interim analysis is planned for the study.

### Methods for additional analyses (e.g. subgroup analyses) {20b}

No additional analyses planned.

### Methods in analysis to handle protocol non-adherence and any statistical methods to handle missing data {20c}

Participants’ completion of questionnaires and MRI data with quality check will be confirmed before commencing the intervention, minimising the risk of missing data at the baseline timepoint. For measures that have missing data for a single time-point we will use listwise deletion for all secondary outcomes except for adverse events and treatment compliance (missing data are not possible), assessment of blinding success and MRI. If there is missing data for the blinding assessment, we will also present the number of participants who either declined to guess which intervention they received, or their selection was not recorded. We will use case-wise deletion for missing data at a single MRI time-point (i.e. if only baseline MRI data was collected, the participant will be excluded from all MRI analyses), and listwise deletion for individual MRI metrics, assuming that data are missing at random and unrelated to measurement of myelin content or lesions in the brain.

### Plans to give access to the full protocol, participant-level data and statistical code {31c}

The published data will be made publicly available via the University of Tasmania’s Research Data Portal. Data availability will be ‘mediated’ allowing anyone to find the data via Research Data Australia and any other RIF-CS compliant search portal. However, access to data will only be available via an email request and access will be contingent on appropriate human research ethics approvals.

All data will be described using RIF-CS metadata consistent with FAIR data principles (Findable, Accessible, Interoperable and Reusable) and will be licensed using a Creative Commons license to ensure appropriate attribution to the data owners. This approach to consistent with sponsor policy, and the Australian Code for the Responsible Conduct of Research.

## Oversight and monitoring

### Composition of the coordinating centre and trial steering committee {5d}

Trial oversight will be the responsibility of the clinical PI and CI (note these two roles are performed by the same individual for this study). The clinical PI/CI will maintain current GCP training and ensure all staff working on the trial are suitably trained and qualified to carry out their delegated duties. The clinical and research PIs will ensure the trial is carried out according to the trial protocol and relevant trial guidelines and regulations.

The clinical PI will ensure appropriate recruitment procedures are in place, ensure eligibility of participant recruitment, manage participant withdrawals and assess, manage and notify SAEs appropriately. They will ensure data privacy and confidentiality is maintained and make data available for monitoring and auditing as required. The Program Manager and research PI will monitor data collection and quality. They will ensure sufficient resources are available to deliver the study and provide staff cover during times of absence. They will have oversight of collected data and manage its release to appropriate members of the team for statistical analysis. The trial management group will meet monthly to discuss day to day management of the trial, consisting of the clinical PI/CI, research PI, trial coordinator, data manager, program manager, research fellows and assistants. They will seek input from the Consumer and Community Reference Committee (quarterly) and Scientific Advisory Committee as required.

The CI will be responsible for all reporting to the sponsor, HMHREC, TGA and funders. The CI or research PI will report on study progress to the MS Research Flagship Leadership Group (monthly), independent Scientific Advisory Committee (bi-annual) and Program Steering Committee (quarterly).

### Composition of the data monitoring committee, its role and reporting structure {21a}

According to National Health and Medical Research Council guidelines [54], no data monitoring committee (DMC) is needed as this is a small phase 1 study.

### Adverse event reporting and harms {22}

#### Assessing

An AE is any untoward medical occurrence in a participant to whom a study intervention has been administered, including occurrences which are not necessarily related to or caused by the intervention. An AE can be any unfavourable or unintended sign, symptom or disease temporarily associated with study activities [54]. AEs will be recorded at each study visit and their severity and causal relationship assessed by the clinical PI. SAEs will be reviewed by an independent neurologist (see below).

#### AE severity assessment

Grade 1—Mild, asymptomatic or mild symptoms, clinical or diagnostic observations only; intervention not indicated.

Grade 2—Moderate, minimal, local or non-invasive interventions indicated; limiting age-appropriate instrumental activity of daily living (ADL)

Grade 3—Severe or medically significant but not immediately life threatening; hospitalisation or prolongation of hospitalisation indicated, disabling, limiting self-care ADL

Grade 4—Life threatening consequences; urgent intervention necessary

Grade 5—Death related to AE

Adverse reactions are those AEs that are deemed by the clinical PI to be related to the study intervention.

- Related—The AE is known to occur with the study intervention, there is a reasonable possibility that the study intervention caused the AE, or there is a temporal relationship between the study intervention and event. Reasonable possibility means that there is evidence to suggest a causal relationship between the study intervention and the AE.
- Not related—There is not a reasonable possibility that the administration of the study intervention caused the event, there is no temporal relationship between the study intervention and event onset, or an alternate aetiology has been established.

A serious adverse event (SAE) is defined as any untoward occurrence that;

- Results in death
- Is life threatening
- Requires hospitalisation or prolongation of existing hospitalisation
- Results in persistent of significant disability or incapacity
- Consists of a congenital abnormality or birth defect
- Is otherwise considered medically significant by the investigator

*Expected AEs:* (common but usually resolve soon after rTMS cessation): Headache; dizziness; tingling of fascial muscles; scalp discomfort.

*Expected SAEs:* admission to hospital for elective surgery; MS relapse; MS disability progression.

Safety oversight will be provided by an independent neurologist, free from the study conduct, who has declared that they are free from conflicts of interest. They will review SAEs and reactions to confirm severity, causality and relationship. A review will be conducted within 3 to 7 days of the event.

#### Reporting

SAEs will be reported to the sponsor within 24 h of becoming aware of the event (except for those listed as expected AEs/SAEs). SUSARs are suspected unexpected serious adverse reactions and these will be reported by the CI to the sponsor and HMHREC within 24 h (i.e. any AE that is serious, unexpected and related). CI to notify Menzies Director within 72 h. The sponsor will notify the Deputy Vice Chancellor of Research and UTAS authority within 72 h.

SUSARs will also be reported to the TGA. If the event is fatal or life threatening, this will be reported within 7 days; all other SUSARs will be reported to the TGA within 15 days.

Events listed as expected AE/SAEs do not require immediate reporting to the sponsor or TGA unless the severity of the event is greater following intervention.

In the event of immediate hazards (requiring immediate actions be taken) to the health and safety of participants, it is the responsibility of the CI to inform the sponsor, HMHREC and TGA of the event within agreed timeframes (24 h if possible and no later than 72 h).

If the trial is temporarily suspended or terminated early due to a safety reason, this will be reported within 15 calendar days.

The CI will send an annual safety report to the HMHREC on the anniversary date of the study approval date. A Development Safety Update Report will also be submitted to the TGA on request.

#### Frequency and plans for auditing trial conduct {23}

There will be no trial monitoring conducted by the sponsor. However, the data will be made available to representatives of the study sponsor, HMHREC or funders as required by law for auditing purposes on request.

Prior to the initiation of the project at the site, the trial master file will be reviewed by the Program Manager to ensure all documents required prior to the commencement of a clinical trial have been obtained.

The Program Manager and research PI will also ensure sufficient staff are available to conduct the study and all have been appropriately trained and have signed the appropriate delegation and training logs.

A final review of the trial master file will be conducted by the Program Manager to ensure study closure and archiving are completed correctly. The Program Manager may conduct regular internal audits (e.g. annually or triggered audits as is necessary).

#### Plans for communicating important protocol amendments to relevant parties (e.g. trial participants, ethical committees) {25}

Ethical review of the study will be performed by the UTAS HMHREC. The study will be conducted in accordance with the International Conference on Harmonisation of Good Clinical Practice guidelines (ICH-GCP) [50], The Privacy Act (1988) [51] and the Australian Code for the Responsible Conduct of Research, 2018 [52] and conditions of HMHREC approval. All amendments will be reviewed by the trial management group and approved by the research PI and CI prior to being submitted to the HMHREC for review. Minor administrative amendments will be implemented immediately and notified. All substantial amendments will require HMHREC review and approval prior to implementation. Any changes in how the study is conducted or new information which may affect the participant’s decision to take part will be discussed with actively enrolled participants and they will be asked to sign a new consent form confirming their decision to continue with the study. The trial registry will also be updated with any substantial amendments.

## Dissemination plans {31a}

Individual results of this study will not be returned to participants. However, a lay summary of results will be communicated via the Menzies newsletter and other avenues. Research findings will be published in open access journals and presented at conferences and seminars, submitted within 12 months following study completion. A final report will be submitted to funding bodies that have awarded grants for the study, as required.

## Discussion

### Protocol development, training and standardisation procedures

Prior to commencing recruitment of participants into the main study, we recruited healthy volunteers to test and standardise rTMS/sham procedures and MRI protocols, ensure researcher training/confidence and confirm site capacity to carry out the procedures according to protocol. For training, two healthy volunteers received both sham and rTMS procedures. Additional healthy volunteers were recruited from Menzies staff and students, who received either a single session of either real rTMS (*n* = 5), or sham (*n* = 3), without additional visits or other assessments. To compare tolerability between two coil locations, participants receiving real rTMS were stimulated with the coil center positioned at different locations on their left and right sides (2cm lateral and 3cm posterior vs. 2cm lateral and aligned with vertex on anterior-posterior axis). All participants reported that the stimulation was equally tolerable at both positions, which informed our choice of coil position. As a pre-trial assessment for the potential for unblinding, rTMS-naïve volunteers receiving sham were asked whether they believed they received rTMS or sham. All participants receiving sham reported that they were unsure but guessed what they think they received: two guessed incorrectly that they were receiving rTMS, and one guessed correctly that they received sham.

We also recruited 2 volunteers to test the MRI acquisition sequence and ensure that the MRI evaluations and imaging parameters were correctly established. Volunteers were staff or students from the Menzies Institute for Medical Research, who gave informed consent and passed the eligibility and safety screening criteria. The healthy volunteer data were used to inform and confirm the main study protocol and will not be analysed with the main study data.

### Operational issues and modifications in response to COVID-19

COVID-19 and any future global pandemics are a new risk that need to be considered for both researchers and participants. All participants will be counselled about COVID-19 symptoms and risks and asked not to attend study visits if they have any COVID-19 symptoms. They will be screened for COVID-19 symptoms at the entrance to the Menzies each day. Researchers and participants will wear personal protective gear to reduce risk. Physical distancing will be maintained as much as possible, with the exception of the 6 min required to deliver the study intervention. Participants reporting or displaying COVID-19 symptoms will be asked to have the COVID-19 test. If they test positive, they may be withdrawn from the study intervention and MRI follow-ups; we will complete study questionnaires and assessments remotely (via phone or online). They may be replaced in the study by a new participant recruited. If the participant tests negative, they will remain in the study and be allowed to return to continue with study visits. As the COVID-19 situation develops, we will take urgent safety measures as required to ensure the safety of participants and researchers are upheld at all times, following government and local guidelines.

### Protocol strengths and limitations

Our study design does not include healthy control participants. We believe this approach is appropriate for a phase 1 safety trial, as previous studies have delivered similar rTMS protocols to healthy and other clinical populations (reviewed in [14, 16]). Moreover, the participant-reported symptom assessments have been validated for use in people living with MS, and possible ceiling effects could limit the comparisons that could be made with healthy participants. Without healthy participants for direct comparison, interpretation of the direction and scale of changes in some MRI measures, such as DTI, may be limited. However, we mitigate this possible limitation by interpreting results with reference to public databases and published healthy control norms.

Our randomised sham-controlled design allows us to assess group differences as well as within-participant change from baseline measures; however, the small sample size does not allow statistical methods to account for individual trajectories over time, or potential effect differences that relate to baseline measures. If the trial progresses to a phase 2 multi-site trial, we anticipate recruitment of a sample that would be sufficient for performing such analyses. This will be particularly important in informing future studies, as the effectiveness of rTMS for remyelination may strongly depend on stage of MS disease progression at the time the intervention is applied (e.g. capacity for remyelination would be limited if substantial axon degeneration has already occurred).

## Trial status

Active, Version 5_01 November 2021. First participant recruited Jan 2021. Twenty participants recruited as of October 2021. Expected last participant, last visit by end December 2021. We originally planned to recruit 30 participants; however, due to restrictions related to the COVID-19 pandemic, the study timeframe was truncated. To meet funding-related timelines, recruitment has now been closed at 20 participants. The protocol manuscript was not submitted earlier because recruitment was previously planned to continue to reach 30 participants. At submission, no data have been statistically analysed for intervention comparisons.

## Data Availability

The study sponsor, clinical PI/CI, research PI and research investigators will have access to the final trial dataset. Research investigators performing analysis will only have access to the de-identified dataset. There are no contractual agreements that limit access to investigators.

## Abbreviations

AE: Adverse event
ANOVA: Analysis of variance
ANZCTR: Australia and New Zealand Clinical Trial Registry
AQoL-8D: Assessment of Quality of Life 8 Dimensions
CI: Chief investigator
DMR: Digital Medical Record
DTI: Diffusion tensor imaging
eCRF: Electronic case report form
e-SDMT: Electronic Symbol Digit Modalities Test
EDSS: Expanded Disability Status Scale
FSS: Fatigue Severity Score
GCP: Good clinical practice
HADS: Hospital Anxiety and Depression Scale
HMHREC: Health and Medical Human Research Ethics Committee
iTBS: Intermittent theta-burst stimulation
rTMS: Repetitive transcranial magnetic stimulation
MANOVA: Multivariate analysis of variance
MRI: Magnetic resonance imaging
MS: Multiple sclerosis
MTR: Magnetisation transfer ratio
NHMRC: National Health and Medical Research Council
PI: Principal investigator
PROM: Participant-reported outcome measure
SAE: Serious adverse event
SNAC: Sydney Neuroimaging Analysis Centre
SOC: System Organ Class
SUSARs: Suspected Unexpected Serious Adverse Reaction
TGA: Therapeutic Goods Administration
UTAS: University of Tasmania
VAS: Visual Analogue Scale
